# Comparison of the Bactericidal Effect of Ultrasonic and Heat Combined with Ultrasonic Treatments on Egg Liquids and Additional Analysis of Their Effect by NIR Spectral Analysis

**DOI:** 10.3390/s24144547

**Published:** 2024-07-13

**Authors:** Dávid Nagy, Tamás Zsom, Andrea Taczman-Brückner, Tamás Somogyi, Viktória Zsom-Muha, József Felföldi

**Affiliations:** 1 Department of Food Measurement and Process Control, Institute of Food Science and Technology, Hungarian University of Agriculture and Life Sciences, Somlói út 14-16., H-1118 Budapest, Hungary; 2 Department of Postharvest, Supply Chain, Commerce and Sensory Science, Institute of Food Science and Technology, Hungarian University of Agriculture and Life Sciences, Ménesi út 43-45., H-1118 Budapest, Hungary; 3 Department of Food Microbiology, Hygiene and Safety, Institute of Food Science and Technology, Hungarian University of Agriculture and Life Sciences, Somlói út 14-16., H-1118 Budapest, Hungary

**Keywords:** egg yolk, egg white, whole egg, food safety, *E. coli*, disinfection

## Abstract

Eggs are a valuable source of nutrients, but they represent a food safety risk due to the presence of microbes. In this work, three types of egg liquids (albumen, yolk and whole egg) previously contaminated with *E. coli* were treated with ultrasound (US) and a combination of ultrasound and low (55 °C) temperature (US+H). The US treatment parameters were 20 and 40 kHz and 180 and 300 W power and a 30, 45 or 60 min treatment time. The ultrasonic treatment alone resulted in a reduction in the microbial count of less than 1 log CFU, while the US+H treatment resulted in a reduction in CFU counts to below detectable levels in all three egg liquids. Heat treatment and ultrasound treatment had a synergistic effect on *E. coli* reduction. For all measurements, except for the whole egg samples treated with US, the 20 kHz treated samples showed a significantly (>90% probability level) lower bactericidal effect than the 40 kHz treated samples. PCA and aquaphotometric analysis of NIR spectra showed significant differences between the heat-treated groups’ (H and US+H) and the non-heat-treated groups’ (US and control) NIR spectra. LDA results show that heat-treated groups are distinguishable from non-heat-treated groups (for albumen 91% and for egg yolk and whole egg 100%).

## 1. Introduction

Eggs are a valuable source of protein and nutrients, and their consumption provides nutritional and health benefits [1]. Eggs are widely used as a food ingredient and have several techno-functional properties (e.g., lecithin in the egg yolk—emulsifying effect; egg white—foaming properties) that can significantly contribute to the texture and sensory properties of the product [2]. Shell eggs are fragile, transport and storage can be problematic, and the native form of eggs in particular can carry food-borne pathogens, so liquid egg products are increasingly being used as a substitute for shell eggs [3]. Liquid egg products are widely used by restaurants and various food manufacturers such as confectioners, raw and dry pasta producers, bakeries, etc., because they are much more easy to handle [4]. It is particularly advantageous to use egg liquid when only the yolk or white is needed for production.

The microbiological contamination of eggs comes mainly from the egg shell. The egg shell is contaminated with faeces containing enteric microorganisms such as *Escherichia coli* and *Salmonella spp*. belonging to the *Enterobacteriaceae* family. Microorganisms present in the nest (e.g., *Staphylococcus aureus*) can cause contamination of the shell surface [5]. When the egg is broken, microbes enter the egg liquid. Eggs are rich in proteins [6] and nutrients, which provide a favorable environment for the growth of microorganisms, including food-borne pathogens, posing an increasing risk to food safety. These bacteria can cause nausea, diarrhea and abdominal pain, etc. [7]. Shiga toxin-producing *E. coli* (STEC) strains can cause severe food-borne illness. Among these, *E. coli* O157:H7 is the most important STEC serotype. It can be transmitted to humans mainly through the consumption of STEC-contaminated food such as raw or uncooked meat, milk or even raw egg products [8]. The *Enterobacteriaceae* limits for egg products (at the end of the manufacturing process) are between 10 and 100 CFU/g or ml according to the European Commission Regulation (EC) No. 2073/2005 on microbiological criteria for foodstuffs [9].

The rapid multiplication of these microorganisms not only increases the risk of disease, but also significantly reduces the shelf life of the egg liquid. It is therefore essential to treat the egg liquids with some form of treatment that reduces the initial microbiological load. The most widely used and well known traditional method of treating liquid egg is pasteurization. The product is exposed to heat in order to reduce the concentration of microorganisms to a safe level and at the same time to increase its shelf life. In industrial practice, heat treatment may be combined with the addition of chemical preservatives (e.g., potassium sorbate) to achieve the required microbiological safety level. Commercially available pasteurized egg liquids stored at 4 °C have a shelf life of only 2–3 weeks [3]. However, during pasteurization, proteins are denatured, which negatively affects the physicochemical and functional properties of eggs, including gelling, emulsification and foaming properties [3]. Therefore, scientific research is focusing on new techniques that can effectively reduce the number of microorganisms and more satisfactorily preserve the original nutritional value of the product. Such possible alternative techniques are, for example, the application of ultrasonic treatment, high-hydrostatic pressure treatment and pulsed electric field treatment.

Ultrasonic treatment, as a non-thermal physical treatment method, has applications in many areas of the food industry, such as meat curing, extraction, drying and emulsification [10]. During ultrasonic treatment, the treated material is subjected to high energy and thermal stress, due to the cavitation [11]. Based on electron microscopic analysis, ultrasonic treatment damages microbes at the cellular level (pore formation, cell wall thinning, cell membrane disruption, release of cytoplasmic contents and damage to DNA structure [12,13]). On the other hand, this cell-level destruction is not uniformly observed for all cells [14], because cavitation-induced bubble collapse is concentrated in a small area, and the damaging effect depends on how close the collapse is to the cell itself [12]. The degree of the microbial damage depends, for example, on the ultrasound parameters (intensity and time) [15], the temperature of the medium [16] and the type of microorganism [17]. Meena and co-workers (2024) found that low-intensity ultrasound treatment can damage microbes to an extent that can still be repaired by the organism itself. This effect alters the metabolism of the cells and accelerates their growth rate [18]. These positive results can be beneficially used, for example, in fermentation, where the application of the ultrasound treatment can increase the growth of lactobacilli, thus reducing the fermentation time [19]. However, once a certain limit is reached, the microbes are damaged in a way that leads to their ultimate death. Therefore, ultrasound has become increasingly popular as an alternative disinfection method in the food industry, with the primary aim of disrupting bacterial homeostatic mechanisms [20]. It is also important to mention that in addition to the mechanical effects, ultrasound also has a chemical effect on the inactivation of microbes. The collapse of the bubbles formed by cavitation causes the chemical breakdown of water, producing free radicals (OH^−^, H^+^, H_3_O^+^) that affect the fluidity, permeability and the degradation of the membrane [21] and the DNA of the bacterial cell [13,20]. These effects make ultrasonic treatment suitable for reducing the microbial count in both solid and liquid foods. For example, ultrasound can be used to eliminate microbes and enzymes on the surface of meat [22], thereby increasing its potential shelf life, while softening the texture of the meat [23]. It has also been reported that ultrasound can be used to reduce the microbial count of seafood [24]. According to Cabeza (2005), ultrasound combined with heat treatment reduces the number of *Salmonella enterica* serovar Senftenberg on the surface of egg shells [25]. Duarte et al. (2018) [26] found that ultrasound treatment combined with sodium dichloroisocyanurate reduces microbial contamination of purple cabbage without the loss of physicochemical and sensory characteristics and anthocyanin content. Liquid foods have also been tested. In the case of milk, ultrasound in combination with heat was successfully used to reduce the number of aerobic mesophilic heterotrophic bacteria present and to enzymatically inactivate them [27]. In the case of whole egg liquid, high-intensity ultrasound treatment was found to be effective in reducing *S. enteritidis* contamination, but the treatment was not found to be fully effective [28]. In the case of adajamir juice, shelf life can be extended by the combined treatment of ultrasound and heat, which effectively reduces the concentration of aerobic microbes, yeasts and molds in the sample [29].

Nowadays, non-destructive measurements are becoming increasingly important as they allow a given crop to be tested without damaging it, thus reducing losses during testing [30]. Non-destructive measurements include near-infrared spectroscopy, which is easy to use, fast and inexpensive, and small sample sizes are sufficient for measurements. An additional advantage is that it requires no sample preparation and can be operated on-line. It is particularly effective for process monitoring and quality control [31,32]. It is therefore not surprising that it has been applied to several horticultural crops in order to monitor changes in crop characteristics. For example, it has been used to predict the firmness and sugar content of cherries [33] or even to classify stone fruits based on soluble solid content [34]. On the other hand, NIR spectroscopy is not only useful for horticultural crops, but has also been shown to be useful for analyzing the freshness of shell eggs [35], assessing the quality of olive oil [36] or detecting food adulteration with cassava flour in yoghurts with high efficiency [37]. This method also allows us to analyze the composition of individual materials. Diaz-Olivares et al. (2020) [38] were able to apply NIR spectroscopy to milk analysis to predict the composition of raw milk. In addition, this method has been used to obtain information on the chemical composition of honey, which can be useful in identifying different floral and geographical sources of honey [39]. NIR can also be used to determine the quality of egg yolks, in particular the polyunsaturated fatty acid content, which is important in the analysis of omega-3-enriched eggs, and it is also a suitable technique for the rapid screening of egg yolk samples from different feeding areas [40].

It is important to study these new processing methods to understand their effects and limitations in order to make them suitable for industrial application.

The aim of this work was to compare the bactericidal effect of ultrasonic treatment and low temperature combined with ultrasonic treatment with different parameters on liquid egg products (egg white, egg yolk and whole egg, respectively) artificially contaminated with *E. coli*. A further aim was to investigate the effect of the different treatments on the structure of the egg liquid using NIR spectroscopy and to analyze how the NIR spectra of the egg products change as a result of the treatments.

## 2. Materials and Methods

Materials: Three types of commercially available liquid egg products (manufacturer Capriovus Kft., Szigetcsep, Hungary) were used for the measurements: egg white, egg yolk and whole egg liquid. All three egg liquids were packaged in 1 kg polyethylene-coated cartons. The products were pasteurized and treated with a preservative (potassium sorbate) to ensure a shelf life of 21 days when stored at 0–4 °C. Samples were stored in a refrigerator at the specified temperature range until use in the experiment. In order to achieve good homogeneity of the samples, the appropriate amount of egg liquid per sample type was gently mixed prior to measurement.

### 2.1. Ultrasonic Treatment

An HBM Machines (MJ Mooedrecht, The Netherlands) device was used for ultrasound treatment. The device was two-thirds filled with tap water to provide a medium for ultrasound propagation. Ultrasonic treatment causes an increase in the temperature of the medium [41], so the water in the treatment chamber was tempered to ensure the correct temperature. The temperature was maintained at 18 ± 2 °C for the ultrasound treatment alone and 55 ± 2 °C for the combined treatment. For both measurements, two different frequencies of 20 and 40 kHz and different power levels of 180 W (0.28 W/cm^2^) and 300 W (0.48 W/cm^2^) were used. Treatment duration was 0 (control), 30, 45 and 60 min for ultrasound and 0, 30 and 60 min for combined treatment measurements. For the combined treatment, an additional control sample was also prepared in which only heat stress (without ultrasound treatment) was applied.

Previous measurements [42] showed that the actual absorbed power at both frequencies was 3.7 ± 0.1 W for the device power of 180 W and 6.9 ± 0.1 W for the power of 300 W, based on the operating parameters of the device. The energy dose of the treatment can be calculated by multiplying the treatment time by the absorbed power.

For sample preparation, 180 mL of egg juices was placed in 200 mL glass jars, sealed, and then placed in the treatment chamber. The treated samples were fully submerged under water during the ultrasonic treatment.

**Figure 1:**
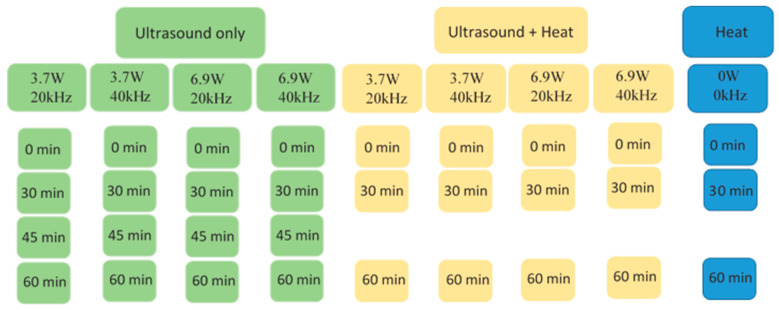
Treatment groups of microbiological measurements.

For both the ultrasound and the combined treatments, five groups were created per egg liquid (Figure 1) based on the combination of treatment parameters (control, 20 kHz and 180 W, 20 kHz and 300 W, 40 kHz and 180 W and 40 kHz and 300 W). The treated group was further divided into three subgroups (30, 45 and 60 min) in the case of ultrasound treatment alone, and two subgroups (30 and 60 min) in the case of combined treatment, depending on the treatment duration. Three replicates of each group were measured.

### 2.2. NIR Measurement

A MetriNIR Research desktop spectrometer (Metrika Kft., Budapest, Hungary) was used for the NIR measurements. Measurements were performed in a transflective configuration in the wavelength range 740 to 1700 nm with a resolution of 2 nm. A water-cooled cuvette with a path length of 0.4 mm was used to avoid temperature effects on the results. The measurement temperature was set at 18 °C. The NIR spectra of 4 parallel samples from each measurement group (Table 1) were measured in a random order, with 4 replicate spectra per sample. In total, more than 700 samples were measured for both treatments, i.e., more than 2500 spectra were analyzed.

For the NIR measurements, 18 mL of treated egg liquid was measured and diluted with 162 mL of distilled water. This resulted in 10 *v*/*v*% solutions. This sample was also used for the aquaphotometric measurements based on the research of Tsenkova et al. (2009) [43]. According to this, the spectral region between 1300 and 1600 nm contains the 12 most important water matrix absorbance coordinates (WAMACs, C1–C12), which can be used for data analysis. Aquagrams were used to visualize the spectral patterns in our data set. A classic aquagram is a star chart that displays the normalized absorbance values of selected water bands, calculated by the following equation:

A’_λ_ = (A_λ_ − μ_λ_)/σ_λ_ (1)

where A’_λ_ is the normalized absorbance, A_λ_ is the absorbance after multiplicative scatter correction (MSC), μ_λ_ is the mean of all spectra for the sample group examined after transformation and σ_λ_ is the standard deviation of all spectra for the sample group examined after transformation [44].

### 2.3. E. coli Inoculation

To test the bactericidal effect of the treatments for each group, 180 mL samples of egg liquids were inoculated with *Escherichia coli* (ATCC 25922). An amount of 180 μL of a 1.5 × 10^8^ CFU × ml^−1^ *E. coli* suspension was used for inoculation. According to the manufacturer’s instructions, a selective and differentiation medium (ChromoBio COLIFORM, BioLab, Diagnostics Laboratory Inc., Budapest Hungary) was prepared to determine the concentration of *E. coli* in the sample. After ultrasound and low temperature combined with ultrasound treatments of the egg samples, the concentration of *E. coli* was determined by spread plating. The plates were incubated at 37 °C for 48 h. Three replicates were performed for each measurement group, resulting in a total of 207 microbiological samples. The logarithm of the colony forming units (CFUs) was determined to assess the bactericidal effect of the treatments.

### 2.4. Data Analysis

MS Excel and IBM SPSS statistical software ver. 25 were used to analyze the microbiological measurement data. NIR spectra were analyzed using the RStudio program (version 2022.07.2 Build 576) aquap2 [45] package. A Savitzky–Golay filter (with a second-order polynomial and a window size of 21 data points) was applied for smoothing, followed by Multiplicative Scatter Correction (MSC) for baseline correction. The near infrared (NIR) spectra of the samples were then analyzed using Principal Component Analysis (PCA). To reduce noise (instrumental factor) in the spectra, only the wavelength range from 950 to 1650 nm was considered.

Linear Discriminant Analysis (LDA) was performed on the PCA results to identify the linear combinations of egg parameters that characterized structural changes in the samples during treatments. The class variables represented the treatments (ultrasound treatment: US; ultrasound combined with heat treatment: US+H; and heat treatment: H) of the liquid egg products. Two thirds of the complete data set was used for model building and the predictive accuracy of the model was assessed by triple cross-validation.

## 3. Results

**Figure 2:**
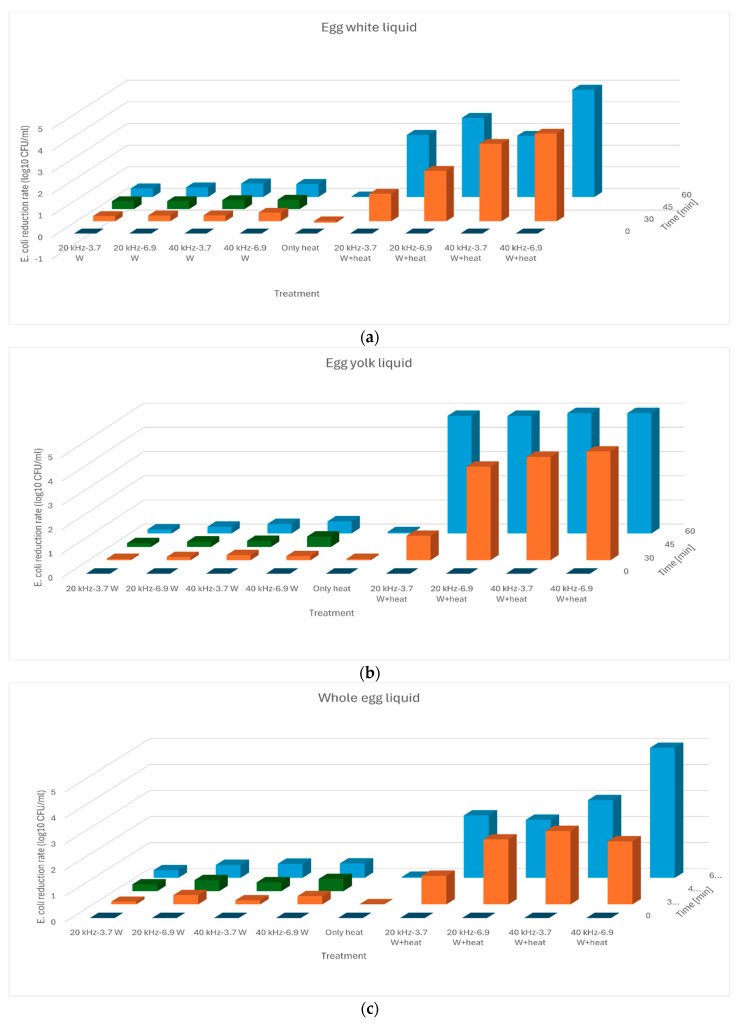
Bactericidal effect of different treatments shown as bactericidal CFU reduction value (log CFU/mL) on egg white liquid (**a**), egg yolk liquid (**b**) and whole egg liquid (**c**).

Both the ultrasonic treatment and the ultrasonic treatment combined with mild heat treatment were bactericidal in terms of the parameters used. The results obtained are shown in Figure 2. The graphs show that the combination of ultrasound and mild heat treatments has a much greater bactericidal effect on *E. coli* than the sample treated with ultrasound alone. Several researchers have found that the use of multiple treatments is beneficial in terms of the microbe killing potential of the method, for example in egg juice (US with lysozyme [46]), in raw milk (US with heat [47]) and in case of salmon (US with blue light [48]). Ultrasound treatment only resulted in a reduction of less than 1 log CFU, even at the highest dose used. In contrast, for the combined treatment, there were treatment settings for all three egg liquids that reduced CFU levels below detectable levels. In this experiment, it was observed that for all three egg liquids, the microbial count was below the detectable limit after 60 min of treatment. In the case of egg yolk, a high level of microbial count reduction was already achieved at 20 kHz with a 3.7 W treatment, whereas in the case of egg white and whole egg liquid, this was only observed at 40 Hz with a 6.9 W treatment level. This may be due to the different composition of egg liquids. Studies suggest that lactose [49] and fat [47] in milk play a role in the inactivation of *Escherichia coli* and *Listeria* species by protecting the bacteria from the microbicidal effects of ultrasound treatment. It was observed that heat treatment alone (55 °C for 30 and 60 min) did not result in a reduction in the CFU values in any type of the egg liquid samples. Thus, heat treatment and ultrasonic treatment have a synergistic bactericidal effect.

**Figure 3:**
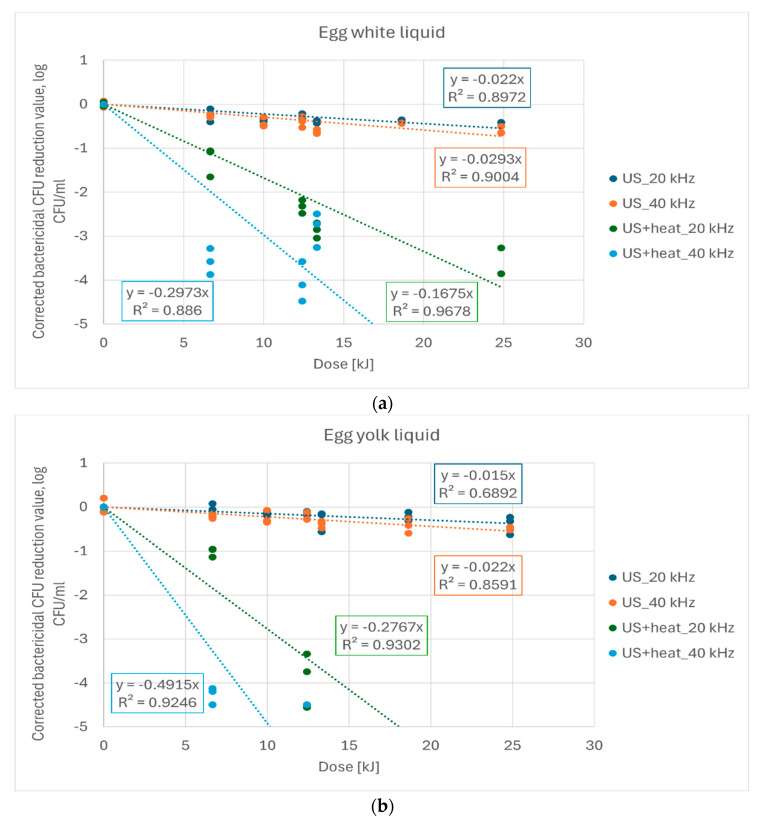

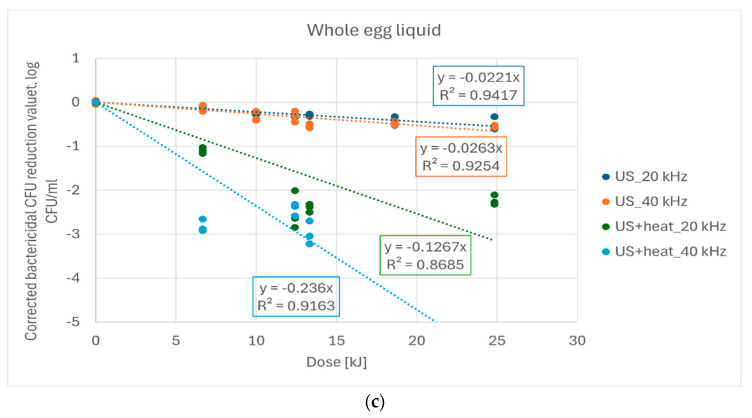
Comparison of the bactericidal effect of ultrasonic treatment and ultrasonic and low temperature combined treatment on egg white (**a**), egg yolk (**b**) and whole egg liquid (**c**).

It can be assumed that the rate of microbe killing is proportional to the energy absorbed (dose). Therefore, the resulting changes were analyzed by linear regression and the resulting statistical parameters were examined. In order to characterize the bactericidal effect, the mean value of the control sample was subtracted from each data series to avoid the effect of initial variability in microbial count, so that the change in microbial count (from 0 to −5) is analyzed as a function of dose. The relationship between dose and reduction in microbial count can be approximated by a linear function. The models are obtained in the form as ‘LogCFU = m × dose’ (‘m’ is negative) so that the intensity of the effect can be clearly described by the slope value. In addition, the higher the absolute value of the slope of the linear equation, the more intense the bactericidal effect. The bactericidal effect of the two treatments as a function of dose is shown in Figure 3.

By analyzing the slope values and their confidence intervals, the following can be concluded: significant differences (*p* < 0.1, one-way ANOVA for slope values with respect to treatment type) were found between all treatments in the case of albumen and egg yolk liquid samples. For whole egg liquid samples, there was no significant difference between the US treatments with respect to frequency, but the combined heat treatment resulted in a significant increase in intensity and a significant difference between the 20 kHz and 40 kHz treatments. The slope of the line (Table 2) describing the bactericidal effect of dose changes is significantly different from zero for both ultrasound alone and the combined ultrasound with low temperature treatment, indicating that ultrasound treatment has a significant effect on reducing microbial count. This was confirmed for all samples and all treatment levels. Ultrasound treatments combined with low temperature had a significantly greater effect on reducing the microbial count at the 99% probability level for all three types of egg liquids than for samples treated with ultrasound alone. For all measurements, except for whole egg samples treated with ultrasound alone, there was a significantly lower bactericidal effect at the minimum 90% probability level for samples treated with 20 kHz than for samples treated with 40 kHz.

Based on the fitted models, the required treatment time for each egg product at a given setting can be predicted to reduce the microbial count below the detectable limit (Table 3).

Based on the fitted models, the US+H treatment at 40 kHz gave the lowest estimated treatment time for all three egg types. For the US only treatment, the expected treatment time to reduce the *E. coli* count below the detectable limit would be very long (413–1502 min), which is not reasonable in an industrial environment. To reduce the treatment time, it is necessary to increase the applied dose of US treatment. However, if the ultrasonic treatment is performed at low temperatures, the treatment time can be significantly reduced (by 87–93%).

**Figure 4:**
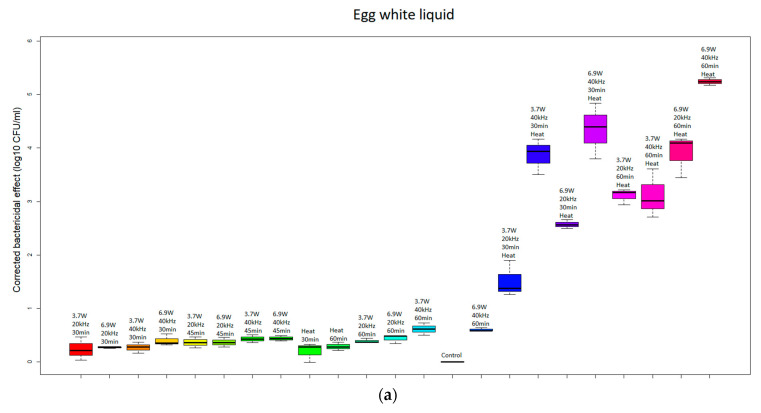

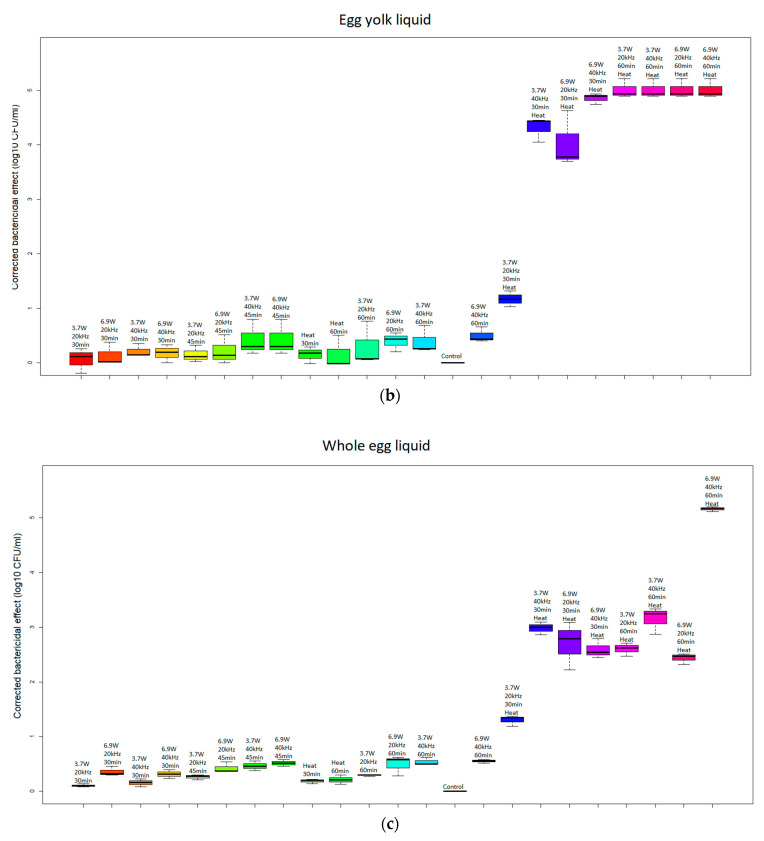
Boxplot of each treatment group based on bactericidal effect (egg white liquid—(**a**); egg yolk liquid—(**b**); and whole egg liquid—(**c**)).

The Tukey post hoc test showed statistically significant differences (*p* < 0.05) in the bactericidal efficacy between the egg products, including egg yolk, egg white and whole egg liquid, treated with the combined treatment of ultrasound and thermal processing, compared with the untreated control samples and the samples treated with either ultrasound or thermal processing alone (Figure 4). Conversely, the samples treated with either thermal or ultrasonic waves alone did not show statistically significant deviations in their bactericidal effects.

### NIR Measurement

Based on the results of our experiment, ultrasound (US) and the combination of ultrasound with mild heat treatment (US+H) showed bactericidal effects. However, it is important to consider that these treatments may induce structural and functional changes in liquid egg products. Near-infrared (NIR) spectroscopy was used to evaluate the changes induced by these treatments.

**Figure 5:**
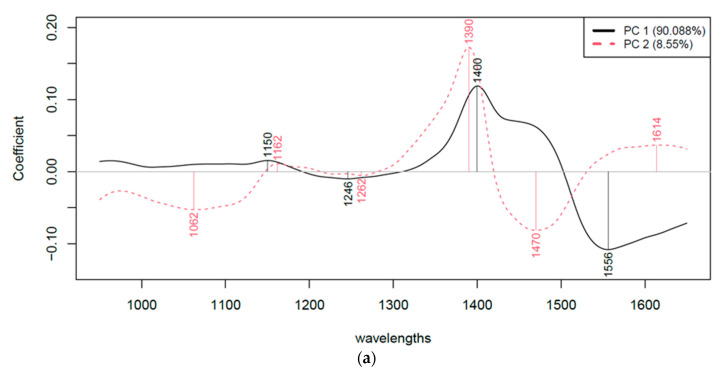

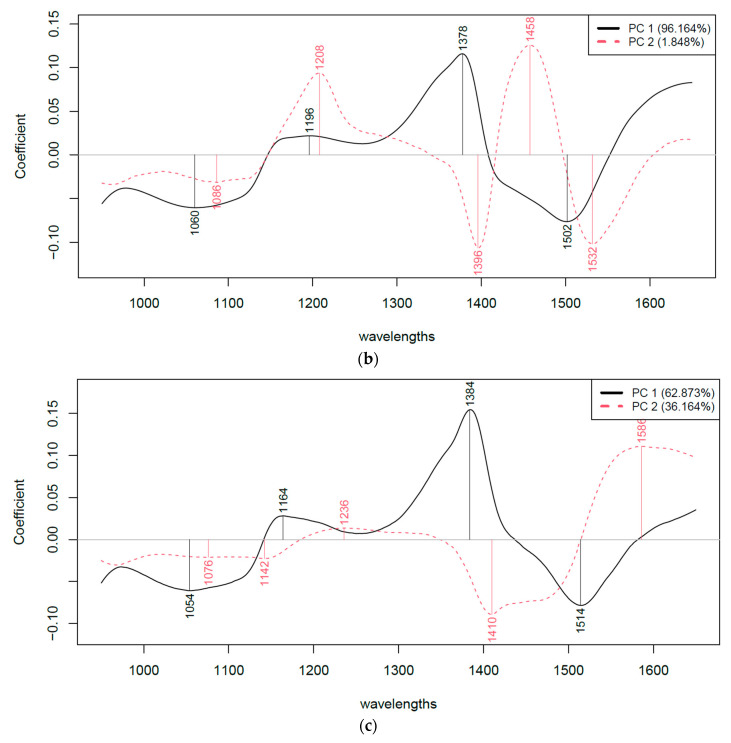
Wavelength contributing most to PC1 and PC2 ((**a**)—liquid egg white; (**b**)—liquid egg yolk; and (**c**)—liquid whole egg).

Principal Component Analysis (PCA) was used to identify the specific wavelengths that contributed most to the formation of the first two principal components, PC1 and PC2 (Figure 5).

In PCA, each principal component is a linear combination of the original variables (in this case, absorbance values at different wavelengths). The coefficients or loadings of this linear combination indicate the relative importance of each variable in defining the principal component. By examining the loadings of PC1 and PC2, which typically account for the majority of the spectral variance, we can identify the wavelengths that are most influential in distinguishing between samples and, by extension, the treatments that have induced these spectral changes [50].

In the context of our ultrasound-treated liquid egg samples, these wavelengths are likely to correspond to the following:

- Water bands: Changes in the 1300–1600 nm range could indicate changes in water structure due to treatments. This range will be further analyzed by aquaphotomics.
- Protein bands: Shifts in N-H (around 1500–1570 nm) or C-H (1100–1250 nm) peaks may reflect protein denaturation or changes in secondary structure.
- Lipid bands: Variations in C-H stretching overtones (1200–1400 nm) may indicate modifications in lipid–protein emulsions [43,51,52,53].

The Principal Component Analysis (PCA) results suggest a notable pattern across all models: the first two principal components, PC1 and PC2, together appear to account for at least 98% of the total spectral variance. In addition, the analysis suggests potentially significant differences between samples treated with heat (US+H, H), those treated with ultrasound alone (US) and the control samples.

**Figure 6:**
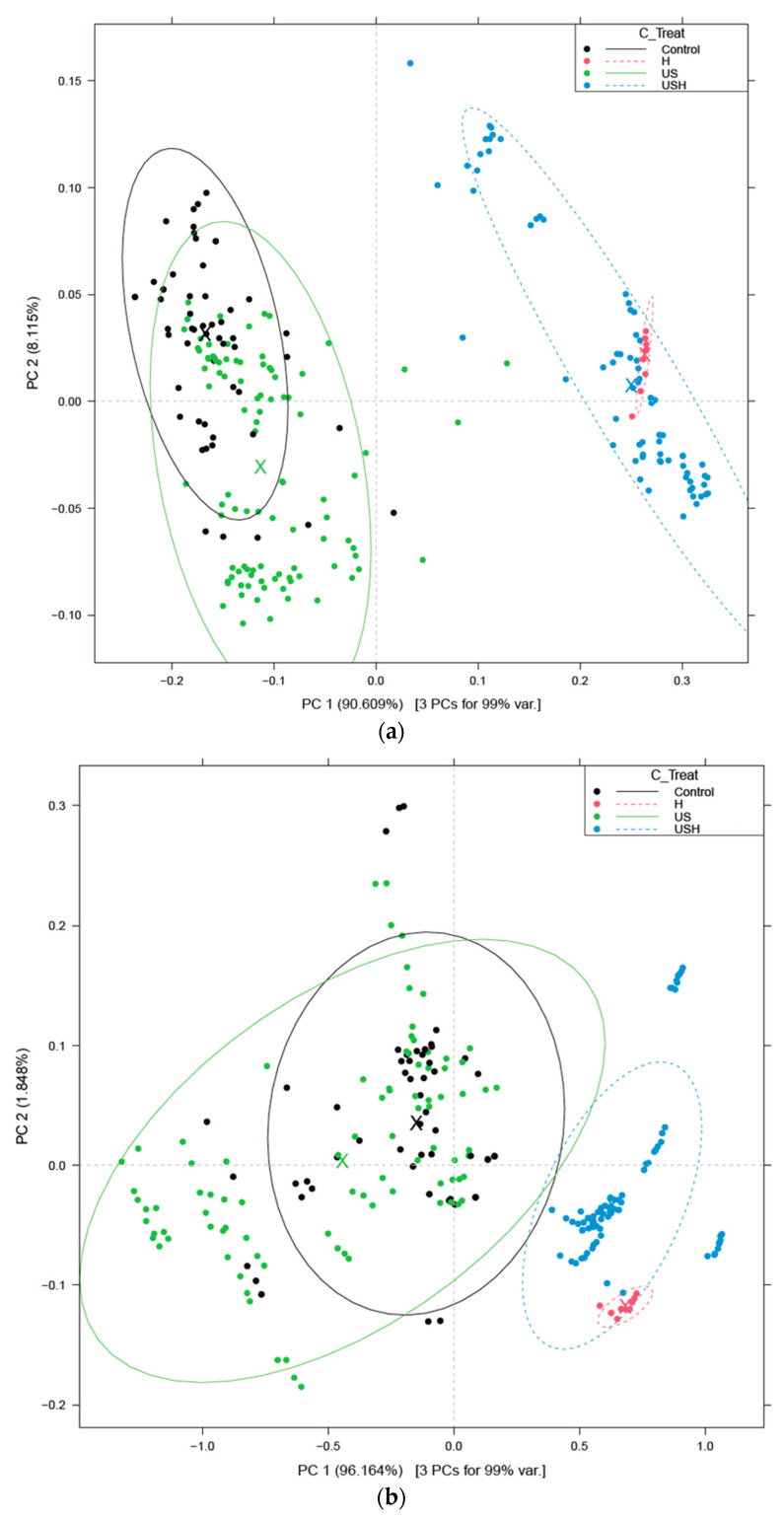

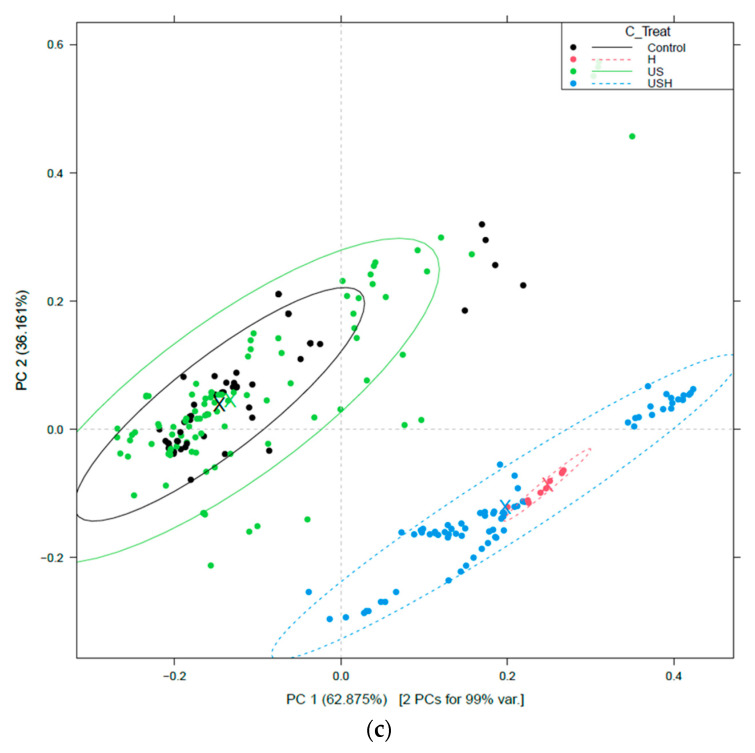
Score plot of PCA on spectral data of egg white liquid (**a**), egg yolk liquid (**b**) and whole egg liquid (**c**) in the range of 950–1630 nm.

The score plots of the PCA models are shown in Figure 6.

While the specific molecular entities that have undergone changes are not identified, our analysis highlights spectral variations in the 1300–1600 nm region, a region dominated by overtones of O-H stretching vibrations. This spectral window is of particular interest because it contains the main water matrix absorption coordinates (WAMACs) used in aquaphotomics [43]. The wavelengths are chosen to observe normalized absorbance values from each WAMAC.

**Figure 7:**
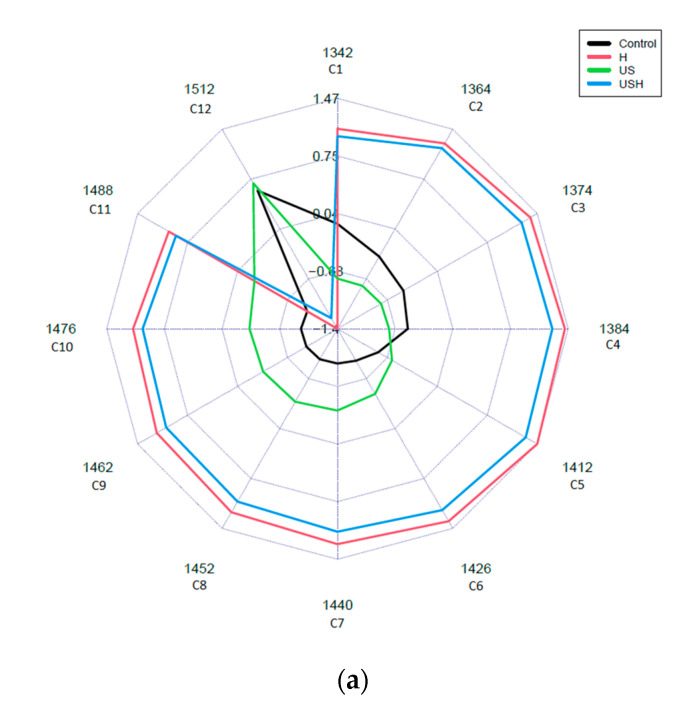

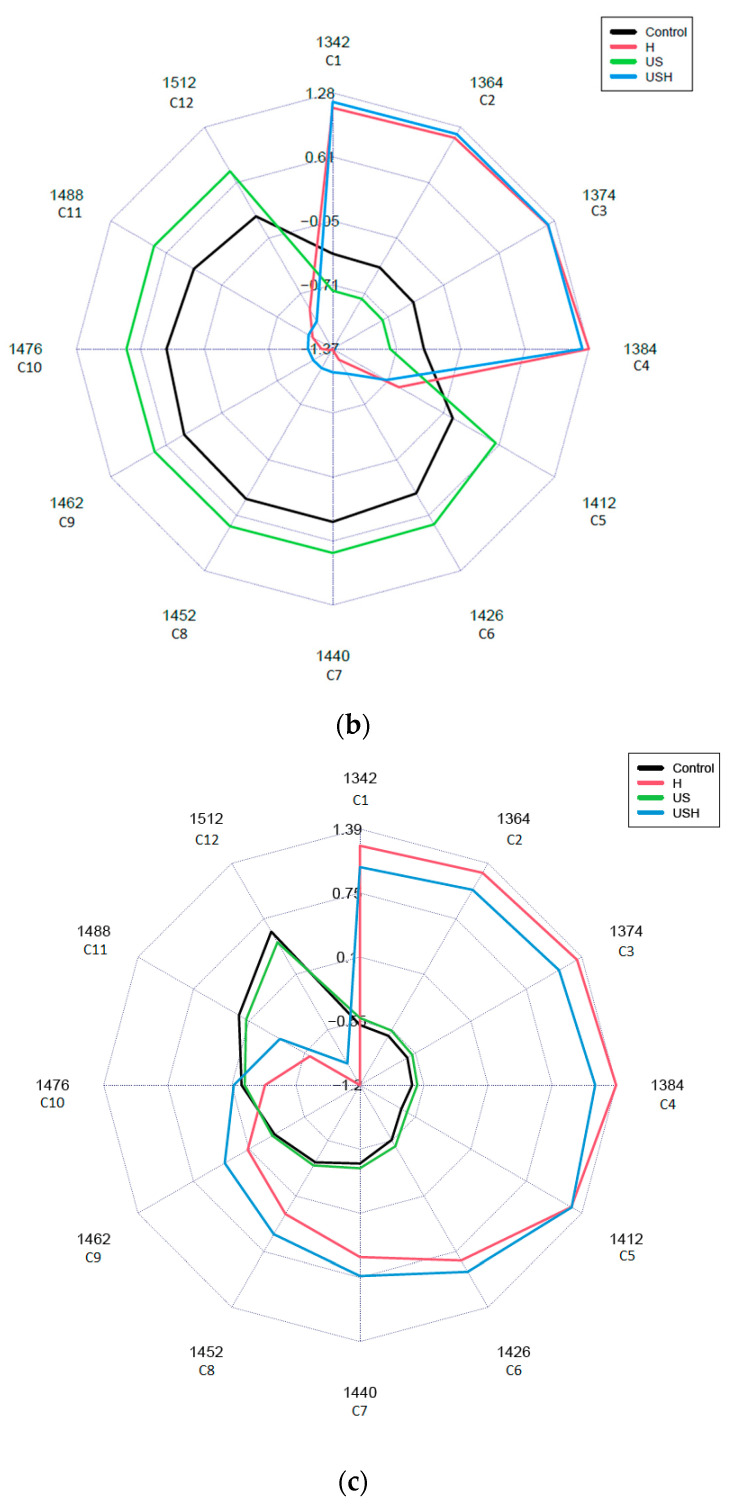
Average normalized absorbance values of the water matrix coordinates of egg white liquid (**a**), egg yolk liquid (**b**) and whole egg liquid (**c**)—aquagrams.

The aquagrams observed are shown in Figure 7.

The aquagrams show distinct patterns, indicating that for WAMACs (Table 4), the patterns are more dependent on the heat treatment than on the ultrasonic treatment, which means that the heat treatment at a low temperature (55 °C) results in a greater change in the spectral properties of the egg liquid than the ultrasonic treatment. Muncan (2019) [51] investigated how the 12 regions of the aquagram (C1–C12) represent changes in the water matrix.

Based on the result of the aquagrams, there are clear differences between the heat- treated (control+heat (H), US+H) and non-heat-treated (the control, US) groups in the case of all egg liquids in all regions (C1–C12), but the different egg liquids show different normalized absorbance patterns. The aquagrams patterns of the egg liquid samples show higher normalized absorbance values in the case of heat treatment in the region C1–C11 for egg white, C1–C5 for egg yolk and C1–C10 for whole egg compared to the patterns of the non-heat-treated groups. For all egg liquids, in the other regions not listed above, the normalized absorbance of the non-heat-treated groups was higher than that of the heat-treated groups. The ultrasound treatment appears to follow the control pattern (note the black and green lines in Figure 6 and Figure 7), indicating a lesser effect on the water bands in the egg samples compared to the heat-treated groups (H and US+H).

Linear Discriminant Analysis (LDA) was performed to classify the samples based on the treatment setups. As previously found, a clear difference was found between US+H and US. The classification accuracy of linear discriminant analysis is shown in Table 5.

The results of the Linear Discriminant Analysis (LDA) indicated that all models exhibited recognition accuracies ranging from 75% to 83% and prediction accuracies between 75% and 80%. A significant difference was observed between the heat-treated groups (H, US+H) and the non-heat-treated groups (US, control), with more than 91% discriminability for egg white and 100% for egg yolk and whole egg liquid. Notwithstanding the observed significant overlap between the ultrasonically treated groups.

## 4. Discussion

Eggs are a valuable source of nutrients and an important food ingredient. However, they also present a food safety risk due to the potential presence of food-borne pathogens. It is necessary to implement a form of treatment to ensure the safe consumption and shelf life of the product. Ultrasound (US) represents an alternative technology that has the potential to reduce the number of microbes in egg liquid, even when this technology is used alone [46,54]. However, the experimental results demonstrate that the combination of ultrasound treatment with heat (US+H) is more effective than ultrasound treatment alone, exhibiting a synergistic bactericidal effect, which corroborates the findings of the hurdle technology. As outlined by Safwa et al. (2023) [20], the combination of ultrasound with other technologies can result in the sensitization of microbial structures to ultrasound waves, which can significantly enhance the degradation and inactivation of microorganisms. The 60 min US+heat treatment at 40 kHz with a power of 6.9 W was found to be an effective method for reducing *E. coli* in all three types of egg liquids (egg white liquid, egg yolk liquid and whole egg liquid) below the detectable limit. It was observed that the treatment had a differential effect on the three types of egg products, which is likely due to their differing compositions. Gera and Doores (2011) [49] observed that the ultrasonic inactivation rates of *E. coli* and *Listeria monocytogenes* differed when inoculated in milk or when inoculated in phosphate buffer. It is important to note that the composition of different egg products necessitates the application of product-specific treatment parameters. The results demonstrate that the 40 kHz treatments (with the exception of the whole egg samples treated only with ultrasound) exhibited a significantly (90% level) higher bactericidal effect than the 20 kHz treatments. This indicates that not only the treatment dose value, but also other setting parameters, influence the inactivation efficiency. The parameters of ultrasonic treatment, including amplitude, wavelength, power and intensity, are of critical importance for the inactivation of bacteria [20].

The NIR spectroscopy results of the egg liquids indicated that the spectra of the heat-treated (control+heat, US+H) and non-heat-treated (US, control) groups were significantly different. The degree of separability was greater than 91% for albumen and 100% for egg yolk and for whole egg liquid. The high degree of accuracy observed in the discrimination of the heat-treated groups provides evidence that heat treatment induces significant and discernible alterations in the NIR spectra of egg samples. It is likely that changes are due to heat-induced protein denaturation, coagulation and alterations in the water structure. Uysal, Boyaci, Soykut and Ertas (2017) [55] observed that as the temperature of the treatment increases, due to denaturation, the solubility of egg white protein decreases, resulting in greater precipitation.

It is noteworthy that the low-heat treatment (55 °C and 60 min without US) employed in this study did not result in a reduction in the microbial count of *E. coli*.

Nevertheless, the considerable overlap between ultrasonically treated samples and control samples does not imply that the ultrasonic treatment has no discernible effect compared to the control. Rather, it indicates that the spectral alterations induced by ultrasonic treatment are less pronounced compared to those induced by thermal treatment. Previous studies [42] have demonstrated significant differences in NIR spectra between ultrasonically treated and control egg samples. Consequently, the lack of significance observed between the ultrasound-treated and control samples in this instance indicates than the US treatment exerts a comparatively minor influence on the observed change, with the effect of low-heat treatment being more pronounced.

Further research is required to elucidate the combined effects of heat and ultrasound treatments on microbial safety and egg quality. The combination of ultrasonic treatment with heat results in a synergistic effect, which allows for a reduction in treatment time or the use of lower temperatures, thus making ultrasound an economically advantageous option for industrial applications. This would contribute to a better understanding of ultrasound’s effects on egg components and its potential role in minimally invasive egg processing strategies.

The results showed that ultrasound treatment can reduce the amount of *E. coli* in egg liquid. In order to use the US treatment in food process industry efficiently, experiments with other microbes in eggs, e.g., *Salmonella* strains, need to be carried out, because the treatment has different effects on different microbes [56]. Ultrasonic technology is now tested in a laboratory environment, but ultrasonic technology has the potential to be used in a flow-through mode, which makes it suitable for integration into the industrial process. Thus, in industrial applications, for example, ultrasonic treatment can be carried out in parallel with heat treatment. Because our experiments have shown that the combined treatment (US+H) is more effective than the individual treatments alone, treatment time can be reduced and treatment at lower temperatures can be successfully applied. The compromise between microbial safety and possible changes in egg quality or functionality should be carefully considered in future processing strategies.

## 5. Conclusions

This study examined the bactericidal effects of ultrasound, combined ultrasound and mild heat (55 °C), and mild heat treatment alone on egg white, egg yolk, and whole egg liquids inoculated with *E. coli*. The relationship between dose and microbial count reduction was approximated by a linear function. The slope of the fitted line describing the bactericidal effect of the dose changes shows that ultrasound treatment alone has a significant microbial count reduction effect, but that the combined treatment has a significantly greater effect on microbial count reduction at the 99% probability level than the ultrasonic treatment alone. For all measurements, except for whole egg samples treated only with ultrasound, there was a significantly lower bactericidal effect of a minimum of 90% probability level for samples treated at 20 kHz than for samples treated at 40 kHz. In the case of ultrasound treatment alone, the treatment time with the settings used in the present experiment should be sufficiently long to reduce the microbial count to an acceptable level. Nevertheless, the treatment time can be considerably reduced (by 87–93%) if the ultrasonic treatment is carried out at a low temperature (55 °C). This indicates a synergistic action of ultrasound and mild heat in enhancing bacterial inactivation.

Near-infrared (NIR) spectroscopy measurements detected differences in the heat-treated samples, including the combined treatment, compared to the untreated control and ultrasound-treated samples. This suggests potential structural or compositional changes induced by the heat component of the treatments.

The experiment showed that US treatment combined with low heat is a promising technique for reducing the number of microbes in egg liquid. However, a number of laboratory tests are still needed before industrial application.

## Figures and Tables

**Table 1:** Treatment groups of NIR measurements.

Group	Absorbed Power [W]	Frequency [kHz]	Time [min]	Temperature [°C]
A	3.7	20	30	18
B	3.7	40	30	18
C	6.9	20	30	18
D	6.9	40	30	18
E	3.7	20	60	18
F	3.7	40	60	18
G	6.9	20	60	18
H	6.9	40	60	18
I	3.7	20	30	55
J	3.7	40	30	55
K	6.9	20	30	55
L	6.9	40	30	55
M	3.7	20	60	55
N	3.7	40	60	55
O	6.9	20	60	55
P	6.9	40	60	55
Heat	0	0	60	55
Control	-	-	-	-

**Table 2:** Slope values (intensity of the bactericidal effect of the treatment).

Treatment	Egg White Liquid	Egg Yolk Liquid	Whole Egg Liquid
US, 20 kHz	−0.0219 ^a^	−0.0149 ^a^	−0.0220 ^a^
US, 40 kHz	−0.0292 ^b^	−0.0220 ^b^	−0.0262 ^a^
US+heat, 20 kHz	−0.1674 ^c^	−0.2766 ^c^	−0.1266 ^b^
US+heat, 40 kHz	−0.2972 ^d^	−0.4914 ^d^	−0.2359 ^c^

a–d: for a given sample type, different letters represent significant differences (*p* < 0.1) between experimental results.

**Table 3:** Based on the fitted models, the treatment time required to reduce the *E. coli* count by five orders of magnitude for each setting.

		3.7 W	6.9 W
**Type**	Setup	Needed treatment time, min	
**Egg white**	US 20 kHz	1023.8	549.0
	US 40 kHz	768.7	412.2
	US+heat 20 kHz	134.5	72.1
	US+heat 40 kHz	75.8	40.6
**Egg yolk**	US 20 kHz	1501.5	805.2
	US 40 kHz	1023.8	549.0
	US+heat 20 kHz	81.4	43.6
	US+heat 40 kHz	45.8	24.6
**Whole egg liquid**	US 20 kHz	1019.1	546.5
	US 40 kHz	856.4	459.2
	US+heat 20 kHz	177.8	95.3
	US+heat 40 kHz	95.4	51.2

**Table 4:** WAMACs [51].

WAMACS	Wavelength	
C1	1336–1348	H_2_O asymmetric stretching vibration
C2	1360–1366	Water solvation shell
C3	1370–1376	H_2_O symmetrical stretching vibration and H_2_O asymmetric stretching vibration
C4	1380–1388	OH-(H_2_O)_1,4_: Water solvation shell O_2_-(H_2_O)_4_: Hydrated superoxide clusters H_2_O symmetrical stretching vibration
C5	1398–1418	Water confined in a local field of ions (trapped water) S0: Free water Water with free OH^−^
C6	1421–1430	Water hydration band
C7	1432–1444	Water molecules with 1 hydrogen bond
C8	1448–1454	Water solvation shell
C9	1458–1468	Water molecules with 2 hydrogen bonds
C10	1472–1482	Water molecules with 3 hydrogen bonds
C11	1482–1495	Water molecules with 4 hydrogen bonds
C12	1506–1516	H_2_O symmetrical stretching vibration Strongly bound water

**Table 5:** LDA values (%) of egg liquid products.

Egg White Liquid																		
	Control	A_US	B_US	C_US	D_US	E_US	F_US	G_US	H_US	L_US+H	P_US+H	O_US+H	K_US+H	J_US+H	N_US+H	I_US+H	M_US+H	Heat
Control	33.38	29.12	16.62	0.87	8.36	0	0	0	0	0	0	0	0	0	0	0	0	0
A_US	16.62	45.88	0	7.03	16.6	0	0	0	0	0	0	0	0	0	0	0	0	0
B_US	25	0	45.88	5.26	12.48	0	0	0	0	0	0	0	0	0	0	0	0	0
C_US	25	25	33.38	86.84	20.85	0	0	0	0	0	0	0	0	0	0	0	0	0
D_US	0	0	4.12	0	20.85	0	0	0	0	0	0	0	0	0	0	0	0	0
E_US	0	0	0	0	0	70.88	0	0	0	0	0	0	0	0	0	0	0	0
F_US	0	0	0	0	4.12	0	100	0	0	0	0	0	0	0	0	0	0	0
G_US	0	0	0	0	0	29.12	0	100	0	0	0	0	0	0	0	0	0	0
H_US	0	0	0	0	8.36	0	0	0	100	0	0	0	0	0	0	0	0	0
L_US+H	0	0	0	0	0	0	0	0	0	100	5.5	0	0	0	0	0	0	0
P_US+H	0	0	0	0	0	0	0	0	0	0	94.5	0	0	0	0	0	0	0
O_US+H	0	0	0	0	8.36	0	0	0	0	0	0	100	0	0	0	0	0	0
K_US+H	0	0	0	0	0	0	0	0	0	0	0	0	100	0	0	0	0	0
J_US+H	0	0	0	0	0	0	0	0	0	0	0	0	0	100	0	0	0	0
N_US+H	0	0	0	0	0	0	0	0	0	0	0	0	0	0	100	0	0	0
I_US+H	0	0	0	0	0	0	0	0	0	0	0	0	0	0	0	100	0	0
M_US+H	0	0	0	0	0	0	0	0	0	0	0	0	0	0	0	0	100	0
Heat	0	0	0	0	0	0	0	0	0	0	0	0	0	0	0	0	0	100
Egg yolk liquid																		
	Control	A_US	B_US	C_US	D_US	E_US	F_US	G_US	H_US	L_US+H	P_US+H	O_US+H	K_US+H	J_US+H	N_US+H	I_US+H	M_US+H	Heat
Control	58.3	0	0	18.42	0	0	0	0	0	0	0	0	0	0	0	0	0	0
A_US	0	50	0	0	0	0	0	0	0	0	0	0	0	0	0	0	0	0
B_US	0	0	58.38	5.26	0	0	0	0	0	0	0	0	0	0	0	0	0	0
C_US	24.97	50	41.62	69.29	66.62	0	0	0	0	0	0	0	0	0	0	0	0	0
D_US	8.36	0	0	1.76	25	4.5	0	0	0	0	0	0	0	0	0	0	0	0
E_US	0	0	0	0	0	45.43	33.33	0	4.12	0	0	0	0	0	0	0	0	0
F_US	0	0	0	0	0	40.93	33.33	4.12	0	0	0	0	0	0	0	0	0	0
G_US	0	0	0	1.76	0	9.14	33.33	95.88	0	0	0	0	0	0	0	0	0	0
H_US	8.36	0	0	3.5	8.38	0	0	0	95.88	0	0	0	0	0	0	0	0	0
L_US+H	0	0	0	0	0	0	0	0	0	100	0	0	0	0	0	0	0	0
P_US+H	0	0	0	0	0	0	0	0	0	0	94.5	0	0	0	5.5	0	0	0
O_US+H	0	0	0	0	0	0	0	0	0	0	0	100	0	0	0	0	0	0
K_US+H	0	0	0	0	0	0	0	0	0	0	0	0	100	0	0	0	0	0
J_US+H	0	0	0	0	0	0	0	0	0	0	0	0	0	100	0	0	0	0
N_US+H	0	0	0	0	0	0	0	0	0	0	5.5	0	0	0	94.5	0	0	0
I_US+H	0	0	0	0	0	0	0	0	0	0	0	0	0	0	0	100	0	0
M_US+H	0	0	0	0	0	0	0	0	0	0	0	0	0	0	0	0	100	0
Heat	0	0	0	0	0	0	0	0	0	0	0	0	0	0	0	0	0	100
Whole egg liquid																		
	**Control**	**A_US**	**B_US**	**C_US**	**D_US**	**E_US**	**F_US**	**G_US**	**H_US**	**L_US+H**	**P_US+H**	**O_US+H**	**K_US+H**	**J_US+H**	**N_US+H**	**I_US+H**	**M_US+H**	**Heat**
Control	25	0	0	0.87	0	0	0	0	0	0	0	0	0	0	0	0	0	0
A_US	16.62	29.12	12.5	0.87	8.38	0	0	0	0	0	0	0	0	0	0	0	0	0
B_US	0	4.12	0	0	0	0	0	0	0	0	0	0	0	0	0	0	0	0
C_US	58.38	58.38	75	94.76	66.62	0	0	0	0	0	0	0	0	0	0	0	0	0
D_US	0	0	0	3.5	8.38	0	0	0	0	0	0	0	0	0	0	0	0	0
E_US	0	0	0	0	0	79.12	0	0	8.38	0	0	0	0	0	0	0	0	0
F_US	0	0	0	0	0	0	66.62	0	0	0	0	0	0	0	0	0	0	0
G_US	0	8.38	12.5	0	16.62	0	0	100	0	0	0	0	0	0	0	0	0	0
H_US	0	0	0	0	0	20.88	33.38	0	91.62	0	0	0	0	0	0	0	0	0
L_US+H	0	0	0	0	0	0	0	0	0	88.83	0	0	0	0	0	0	0	0
P_US+H	0	0	0	0	0	0	0	0	0	0	100	0	0	0	0	0	0	0
O_US+H	0	0	0	0	0	0	0	0	0	0	0	100	0	0	0	0	0	0
K_US+H	0	0	0	0	0	0	0	0	0	0	0	0	100	0	0	0	0	0
J_US+H	0	0	0	0	0	0	0	0	0	11.17	0	0	0	100	0	0	0	0
N_US+H	0	0	0	0	0	0	0	0	0	0	0	0	0	0	100	0	0	0
I_US+H	0	0	0	0	0	0	0	0	0	0	0	0	0	0	0	100	0	0
M_US+H	0	0	0	0	0	0	0	0	0	0	0	0	0	0	0	0	100	0
Heat	0	0	0	0	0	0	0	0	0	0	0	0	0	0	0	0	0	100

## Data Availability

The data presented intó this study are available on request from the corresponding author. The data are not publicly available due to this research is still in progress.
